# Real-World Treatment Trends Among Patients with Metastatic Castration-Sensitive Prostate Cancer: Results from an International Study

**DOI:** 10.1093/oncolo/oyad045

**Published:** 2023-04-04

**Authors:** Pedro C Barata, Andrea Leith, Amanda Ribbands, Rachel Montgomery, Matthew Last, Bhakti Arondekar, Jasmina Ivanova, Alexander Niyazov

**Affiliations:** Department of Hematology Oncology, University Hospitals Seidman Cancer Center, Cleveland, OH, USA; Oncology, Adelphi Real World, Bollington, UK; Oncology, Adelphi Real World, Bollington, UK; Oncology, Adelphi Real World, Bollington, UK; Formerly of Adelphi Real World, Bollington, UK; Global Value and Evidence, Pfizer Inc., Collegeville, PA, USA; Global Value and Evidence, Pfizer Inc., New York, NY, USA; Global Value and Evidence, Pfizer Inc., New York, NY, USA

**Keywords:** disease management, prostatic neoplasms/therapy, prostatic neoplasms, castration-resistant/drug therapy, androgen antagonists

## Abstract

**Background:**

Continuous androgen deprivation therapy ± first-generation non-steroidal antiandrogen was previously the standard-of-care for patients with metastatic castration-sensitive prostate cancer (mCSPC). Treatment intensification with novel hormonal therapy (NHT) or taxane chemotherapy is now approved and guideline-recommended for these patients.

**Methods:**

Physician-reported data on adult patients with mCSPC from the Adelphi Prostate Cancer Disease Specific Programme were analyzed descriptively. We evaluated real-world treatment trends for patients with mCSPC in 5 European countries (United Kingdom, France, Germany, Spain, and Italy) and the United States (US), looking at differences between patients initiating treatment in 2016-2018 and in 2019-2020. We also investigated treatment trends by ethnicity and insurance status in the US.

**Results:**

This study found that most patients with mCSPC do not receive treatment intensification. However, greater use of treatment intensification with NHT and taxane chemotherapy was observed in 2019-2020 than in 2016-2018 across 5 European countries. In the US, greater use of treatment intensification with NHT in 2019-2020 than in 2016-2018 was observed for all ethnicity groups and those with Medicare and commercial insurance status.

**Conclusions:**

As the number of patients with mCSPC who receive treatment intensification increases, more patients who progress to metastatic castration-resistant prostate cancer (mCRPC) will have been exposed to intensified treatments. Treatment options for patients with mCSPC and mCRPC overlap, suggesting that an unmet need will emerge for new therapies. Further studies are needed to understand optimal treatment sequencing in mCSPC and mCRPC.

## Teaser

This study evaluated real-world metastatic castration-sensitive prostate cancer (mCSPC) treatment trends between 2016 to 2020 across a large, diverse patient population. The study also investigated whether treatment trends vary by ethnicity and insurance status.

**Implications for Practice**

In this real-world study of adult patients with metastatic castration-sensitive prostate cancer (mCSPC), greater use of treatment intensification with novel hormonal therapy was observed in 2019-2020 than in 2016-2018 across 5 European countries and the United States. Across Europe, greater use of taxane chemotherapy was also observed in the later time period. As the number of patients with mCSPC who receive treatment intensification increases, more patients will have been exposed to a greater number of intensified treatments, suggesting that an unmet need will emerge for new therapies in metastatic castration-resistant prostate cancer.

## Introduction

Continuous androgen deprivation therapy (ADT) with or without a first-generation nonsteroidal antiandrogen (NSAA) is commonly used to treat patients with metastatic ­castration-sensitive prostate cancer (mCSPC) and was previously the standard-of-care for these patients.^1^ When therapies such as docetaxel (taxane chemotherapy), abiraterone, apalutamide, or enzalutamide (ie, novel hormonal therapies [NHTs]) are added to ADT, this is often referred to as treatment intensification.^2^

Pivotal randomized clinical trials of treatment intensification in patients with mCSPC have been published from 2015 onwards, including CHAARTED and STAMPEDE (docetaxel)^3-6^; LATITUDE and STAMPEDE (abiraterone)^7-11^; TITAN (apalutamide)^12-14^; and ARCHES and ENZAMET (enzalutamide).^15,16^ These trials demonstrated improved ­progression-free survival (PFS) and overall survival (OS) compared with placebo in patients with mCSPC treated with ADT. In addition, NHTs demonstrated similar^12-14,17,18^ or even improved^9^ quality-of-life compared with placebo in these patients. Prostate cancer guidelines therefore recommend treatment intensification for patients with mCSPC.^2,19-22^

The timing of different therapy approvals varied between Europe and the United States (US). The European Medicines Agency (EMA) approved abiraterone in October 2017^23^; docetaxel in October 2019^24^; apalutamide in December 2019^25^; and enzalutamide in March 2021^26^ for patients with mCSPC. In the US, the Food and Drug Administration (FDA) approved abiraterone in February 2018^27^; apalutamide in September 2019^28^; and enzalutamide in December 2019^29^ for patients with mCSPC. In the US, use of docetaxel in patients with mCSPC is currently off-label.^30^ However, off-label prescribing is common in oncology^31^ and is evident in real-world studies of patients with mCSPC.^32-43^ Research is also emerging on the use of triplet therapy in mCSPC,^44,45^ and this may already be making its way into real-world practice.

Ultimately, real-world treatment utilization may depend on a number of country-specific and region-specific factors including treatment availability, regulatory approval, reimbursement, and insurance status (particularly in the US),^46,47^ as well as patient preferences and physician perceptions.^48-50^ There are claims-based US and Canadian data showing that most patients do not receive treatment intensification for mCSPC^32-43^; however, it is important to keep in mind that these claims-based studies began before most FDA approvals of NHTs in mCSPC. To our knowledge, no real-world data currently exist on the utilization of mCSPC treatment intensification in Europe.

The objective of our study was to evaluate real-world mCSPC treatment trends from 2016 to 2020 across a large, diverse patient population in 5 European countries (United Kingdom [UK], France, Germany, Spain, and Italy) and the US. We also investigated whether treatment trends vary by ethnicity and insurance status in the US. Our recent time period allows us to capture treatment trends after the approval of NHTs for use in mCSPC.

## Methods

### Study Design

Data from the Adelphi Prostate Cancer Disease Specific Programme (DSP) were utilized and subsequently analyzed for the purpose of this study. The DSP is a point-in-time, ­physician-conducted extraction of medical chart data. The surveys are conducted in routine clinical practice and the data collected describe patient demographics and clinical characteristics, prostate cancer disease management including treatment history, the burden and impact of prostate cancer, and associated treatment effects from the perspective of the physician. The DSP methodology has been previously published and validated.^51-53^

Physicians in the UK, France, Germany, Spain, Italy, and the US reported information for their patients with metastatic prostate cancer attending a physician’s appointment between January and August 2020.

### Participants

A geographically diverse sample of physicians was identified by local fieldwork agents using physician panels and publicly available lists. All physicians self-identified as oncologists or urologists (or prostate/specialist cancer surgeons, who were included as urologists). All physicians had personal responsibility for prescribing decisions for patients with prostate cancer and were seeing 2 or more patients with mCSPC and 2 or more patients with mCRPC per month.

### Patient Inclusion Criteria

At the time of data collection, patients included in this study were males aged ≥18 years, currently diagnosed with metastatic prostate cancer, not known to have ever enrolled in a prostate cancer clinical trial, and were receiving treatment for metastatic prostate cancer (any line). The data we analyzed included patients with mCSPC at the time of data collection or who had mCSPC previously and progressed to metastatic castration-resistant prostate cancer (mCRPC). All patients who initiated an mCSPC treatment between 2016 and 2020 were included in the study.

### Treatments Received

For the purpose of this study, we define ADT as medical castration with or without a first-generation NSAA. Medical ADT included ketoconazole, chlormadinone, cyproterone, buserelin, degarelix, goserelin, leuprorelin, triptorelin, and histrelin. First-generation NSAA included bicalutamide, flutamide, and nilutamide.

In the real-world setting, treatment intensification may include agents that are not currently approved for use in mCSPC. For the purposes of this study, treatment intensification is therefore defined as any other systemic therapy in addition to ADT, including NHT, taxane chemotherapy, ­taxane-based combinations, and other regimens such as radiotherapy and immunotherapy, regardless of the treatment’s indication or approval status. NHTs included abiraterone, apalutamide, darolutamide, and enzalutamide. Taxane chemotherapy included docetaxel, cabazitaxel, and paclitaxel.

### Physician-Reported Data

Participating physicians completed an attitudinal survey with questions on physician and practice characteristics. Following this, physicians completed detailed electronic patient record forms (ePRFs) for their next eligible, adult patients treated for mCSPC (4 patients) or mCRPC (4 patients). The number of patients per physician was limited to allow for a varied representation.

The ePRFs collected detailed information on patient demographics and clinical characteristics, patient management, and treatment history at the time of data collection. Information was collected on patients with mCSPC at data collection as well as patients with mCRPC at data collection who had historical mCSPC treatment information available. Ethnicity was identified by physicians and was not self-identified by patients.

The Eastern Cooperative Oncology Group Performance Status Scale (ECOG) was used to assess performance status, which scores from 0 (fully active) to 4 (completely disabled).^54^

### Ethics

The Adelphi DSP was submitted to and obtained exemption from the Western Institutional Review Board, study protocol number AG8741.

Data collection was undertaken in line with European Pharmaceutical Marketing Research Association guidelines^55^ and as such it did not require ethics committee approval. Each survey was performed in full accordance with relevant legislation at the time of data collection, including the US Health Insurance Portability and Accountability Act 1996,^56^ and the Health Information Technology for Economic and Clinical Health Act.^57^ Data were collected in such a way that patients and physicians could not be identified directly.

### Analysis

Data were analyzed descriptively using IBM SPSS Data Collection Survey Reporter Version 6 or later (International Business Machines Corp., New York, USA). For continuous variables, we reported mean and SD, and/or median and range. For categorical variables, frequency and percentage distribution were reported.

Treatments initiated across all lines of mCSPC therapy in each country were reported based on year of treatment initiation and split into 2 time periods: 2016-2018 and 2019-2020. Our analysis looked at the differences between the 2 time periods. While patients appeared in each time period only once, patients could initiate multiple lines of therapy within each time period. This resulted in some answers totaling more than 100% if different options were chosen across treatment lines.

Time to treatment intensification was calculated only among patients who received treatment intensification in mCSPC and was measured both from the date of mCSPC diagnosis to the initiation of treatment intensification and from the time of first-line mCSPC treatment initiation to the initiation of treatment intensification.

## Results

### Patient Characteristics

Across all countries, 328 physicians (278 in Europe and 50 in the US) provided data on 1560 patients (1321 in Europe and 239 in the US) with mCSPC (Tables 1 and 2). This included patients with mCSPC at the time of data collection and patients with mCRPC at the time of data collection who had historical mCSPC treatment information available.

**Table 1.** Patient baseline demographics in 5 European countries and the US.

	Europe^a^ (*n* = 1321)	US (*n* = 239)
Physician specialty, *n* (%)		
Oncologist	1111 (84)	179 (75)
Medical oncologist	1017 (77)	179 (75)
Radiation oncologist	26 (2)	0 (0)
Clinical oncologist^b^	68 (5)	0 (0)
Urologist^c^	210 (16)	60 (25)
Hospital type, *n* (%)		
Academic/cancer center	705 (53)	114 (48)
Community	616 (47)	125 (52)
Patient age at time of data collection, *n*		
Median (range)	72.0 (45-90)	69.0 (50-90)
Patient age at time of mCSPC diagnosis, *n*		
Total	586	129
Median (range)	70.0 (44-88)	68.0 (49-89)
Family history of prostate cancer, *n* (%)		
Yes	130 (10)	34 (14)
No	1122 (85)	193 (81)
Don’t know	69 (5)	12 (5)
Ethnicity, *n* (%)		
White/Caucasian	1231 (93)	159 (67)
Afro-Caribbean/African American^d^	34 (3)	49 (21)
Other	56 (4)	31 (13)
US geography^e^, *n* (%)		
Midwest	—	39 (16)
Northeast	—	123 (51)
South	—	47 (20)
West	—	30 (13)

^a^Includes 5 European countries only: UK, France, Germany, Spain, and Italy.

^b^Clinical oncologist is a UK-specific specialty covering both medical and radiation specialties.

^c^Urologist includes 7 physicians in Spain who selected “Prostate/specialist cancer surgeon.”.

^d^Afro-Caribbean in Europe; African American in US.

^e^States within each geographic region:.

-Midwest: Indiana, Illinois, Michigan, Ohio, Wisconsin, Iowa, Nebraska, Kansas, North Dakota, Minnesota, South Dakota, and Missouri.

-Northeast: Connecticut, Maine, Massachusetts, New Hampshire, Rhode Island, Vermont, New Jersey, New York, and Pennsylvania.

-South: Delaware, District of Columbia, Florida, Georgia, Maryland, North Carolina, South Carolina, Virginia, West Virginia, Alabama, Kentucky, Mississippi, Tennessee, Arkansas, Louisiana, Oklahoma, and Texas.

-West: Arizona, Colorado, Idaho, New Mexico, Montana, Utah, Nevada, Wyoming, Alaska, California, Hawaii, Oregon, and Washington.

Abbreviation: mCSPC, metastatic castration-sensitive prostate cancer; UK, United Kingdom; US, United States.

**Table 2.** Patient baseline disease characteristics in 5 European countries and the US.

	Europe^a^ (*n* = 1321)	US (*n* = 239)
Disease state at time of data collection, *n* (%)		
mCSPC	849 (64)	146 (61)
mCRPC with mCSPC treatment history	472 (36)	93 (39)
Patients with metastases at time of data collection, *n* (%)		
Bone	1163 (88)	164 (69)
Non-regional/distant lymph nodes	481 (36)	66 (28)
Visceral	256 (19)	71 (30)
* *Liver	96 (7)	16 (7)
Other	10 (1)	4 (2)
ECOG score at mCSPC diagnosis, *n* (%)		
0	353 (27)	60 (25)
1	741 (56)	150 (63)
2	191 (14)	24 (10)
3	33 (2)	2 (1)
4	2 (0)	2 (1)
Unknown/not assessed	1 (0)	1 (0)

^a^Includes 5 European countries only: UK, France, Germany, Spain, and Italy.

Abbreviations: ECOG, Eastern Cooperative Oncology Group; mCSPC, metastatic castration-sensitive prostate cancer; mCRPC, metastatic castration-resistant prostate cancer; UK, United Kingdom; US, United States.

Across the 5 European countries, at the time of mCSPC diagnosis, the median (range) age was 70 (44-88) years. Most patients (83%) had an Eastern Cooperative Oncology Group (ECOG) performance status of 0 or 1 at the time of mCSPC diagnosis. Ninety-three percent of patients were White/Caucasian, 3% were Afro-Caribbean, and 4% were other ethnicities. At the time of data collection, the median (range) age was 72 (45-90) years. Eighty-eight percent of patients had bone metastases and 19% of patients had visceral metastases. Most patients (84%) were treated by an oncologist and 16% were treated by a urologist (including prostate/specialist cancer surgeons). Overall, just under half (47%) of patients were treated at a community hospital and just over half (53%) were treated at an academic medical center. In Germany and Italy, most patients (81% and 64%, respectively) were treated at a community hospital. However, in the UK, France, and Spain, most patients (78%, 57%, and 86%, respectively) were treated at an academic medical center (Supplementary Tables S1A and S1B).

In the US, at the time of mCSPC diagnosis, the median (range) age was 68 (49-89) years. Most patients (88%) had an ECOG performance status of 0 or 1. Sixty-seven percent of US patients were White/Caucasian, 21% were African American, and 13% were other ethnicities (including Hispanic/Latino, Asian, Middle Eastern, and mixed race). Fifty-one percent of US patients were in the Northeast. At the time of data collection, the median (range) age was 69 (50-90) years. Sixty-nine percent of patients had bone metastases and 30% of patients had visceral metastases. Three-quarters (75%) of patients were treated by a medical oncologist and the remaining quarter (25%) was treated by a urologist. Just over half (52%) of patients were treated at a community hospital and just under half (48%) were treated at an academic medical center.

In total, 58% of US patients had Medicare insurance, 33% commercial, 6% Medicaid, and 3% other/no health insurance. A greater percentage of African American patients had Medicare insurance (69%) than White/Caucasian patients or patients of other ethnicity (both 55%), as well as other/no health insurance (8%, 1%, and 3%, for African American, White/Caucasian, and other ethnicity groups, respectively). A smaller percentage of African American patients had commercial insurance (14%) than White/Caucasian patients or patients of other ethnicity (39% and 29%, respectively). A smaller percentage of White/Caucasian patients had Medicaid (4%) than African American patients or patients of other ethnicity (8% and 13%, respectively; Supplementary Table S2).

### Treatment Trends Across 5 European Countries by Year of Initiation

**Figure 1.**
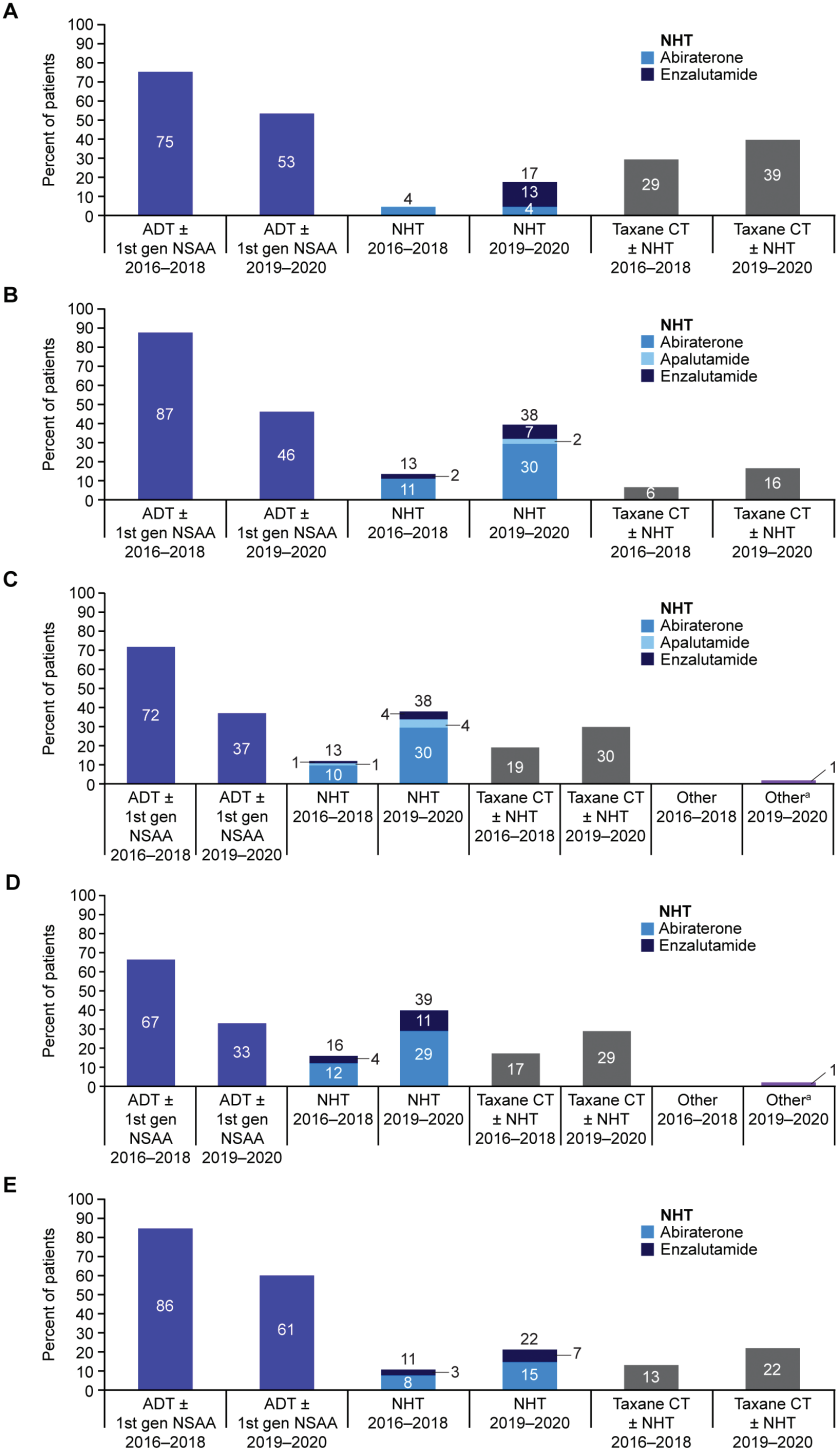
mCSPC treatment trends in 5 European countries by year of treatment initiation. mCSPC treatment trends by year of treatment initiation (**A**), UK [2016-2018 *n* = 56; 2019-2020 *n* = 151]; (**B**) France [2016-2018 *n* = 142; 2019-2020 *n* = 243]; (**C**) Germany [2016-2018 *n* = 88; 2019-2020 *n* = 186]; (**D**) Spain [2016-2018 *n* = 69; 2019-2020 *n* = 160]; (**E**) Italy [2016-2018 *n* = 99; 2019-2020 *n* = 167]). Percentages may add up to >100% as patients could have had multiple treatment lines initiated within the given time period. No patients in this analysis received darolutamide in combination with docetaxel. ^a^Radium-223-containing regimen. Abbreviations: ADT, androgen deprivation therapy; CT, chemotherapy; gen, generation; mCSPC, metastatic castration-sensitive prostate cancer; NHT, novel hormonal therapy; NSAA, nonsteroidal antiandrogen; UK, United Kingdom.

Across the 5 European countries (UK, France, Germany, Spain, and Italy), greater use of treatment intensification (NHT and taxane chemotherapy) for patients with mCSPC was observed in 2019-2020 than in 2016-2018. Germany saw the largest change in the use of treatment intensification and Italy saw the smallest change. The greatest overall use of treatment intensification in 2019-2020 was seen in Spain, with 29% of patients receiving taxane chemotherapy, 29% receiving abiraterone, and 11% receiving enzalutamide. In the UK, treatment intensification with enzalutamide was seen more than treatment intensification with abiraterone. In France, Germany, Spain, and Italy, treatment intensification with abiraterone was seen more than treatment intensification with enzalutamide. The UK had the highest percentage of patients who received treatment intensification with taxane chemotherapy relative to France, Germany, Spain, and Italy (Fig. 1A-1E).

Median (range) time from mCSPC diagnosis to treatment intensification remained at 0 (0-72) months for 2016-2018 and 2019-2020. Mean (SD) time from mCSPC diagnosis to treatment intensification in 2016-2018 was 4.1 (11.0) months, and in 2019-2020 was 3.1 (8.1) months. Median (range) time from first-line mCSPC treatment initiation to treatment intensification remained at 0 (0-69) months for the 2 time periods. Mean (SD) time from first-line mCSPC treatment initiation to treatment intensification in 2016-2018 was 3.1 (9.5) months, and in 2019-2020 was 0.8 (4.8) months (Table 3).

**Table 3.** Time to mCSPC treatment intensification by year of mCSPC treatment initiation.

Year of mCSPC treatment initiation	Europe^a^			US			Total (Europe and US)		
	2016-2020	2016-2018	2019-2020	2016-2020	2016-2018	2019-2020	2016-2020	2016-2018	2019-2020
Time from mCSPC diagnosis to treatment intensification, months									
Total, *n*	202	83	129	58	32	26	260	115	155
Mean	2.7	4.1	3.1	2.9	3.2	2.5	2.7	3.8	3.0
Median	0.0	0.0	0.0	0.0	0.0	0.0	0.0	0.0	0.0
Range	0-72	0-72	0-72	0-30	0-30	0-28	0-72	0-72	0-72
SD	7.8	11.0	8.1	7.0	7.5	6.4	7.6	10.1	7.9
Time from first-line mCSPC treatment initiation to treatment intensification, months									
Total, *n*	634	131	525	148	43	107	782	174	632
Mean	0.7	3.1	0.8	0.4	0.4	0.3	0.7	2.4	0.7
Median (range)	0.0 (0-69)	0.0 (0-69)	0.0 (0-69)	0.0 (0-19)	0.0 (0-19)	0.0 (0-9)	0.0 (0-69)	0.0 (0-69)	0.0 (0-69)
SD	4.6	9.5	4.8	2.0	2.9	1.5	4.2	8.5	4.4

Time to treatment intensification was calculated only among patients who received treatment intensification in mCSPC, and was measured both from the date of mCSPC diagnosis to the initiation of treatment intensification and from the time of first-line mCSPC treatment initiation to the initiation of treatment intensification.

^a^Includes 5 European countries only: UK, France, Germany, Spain, and Italy.

Abbreviation: mCSPC, metastatic castration-sensitive prostate cancer; UK, United Kingdom; US, United States.

### Treatment Trends in the US by Year of Initiation

**Figure 2.**
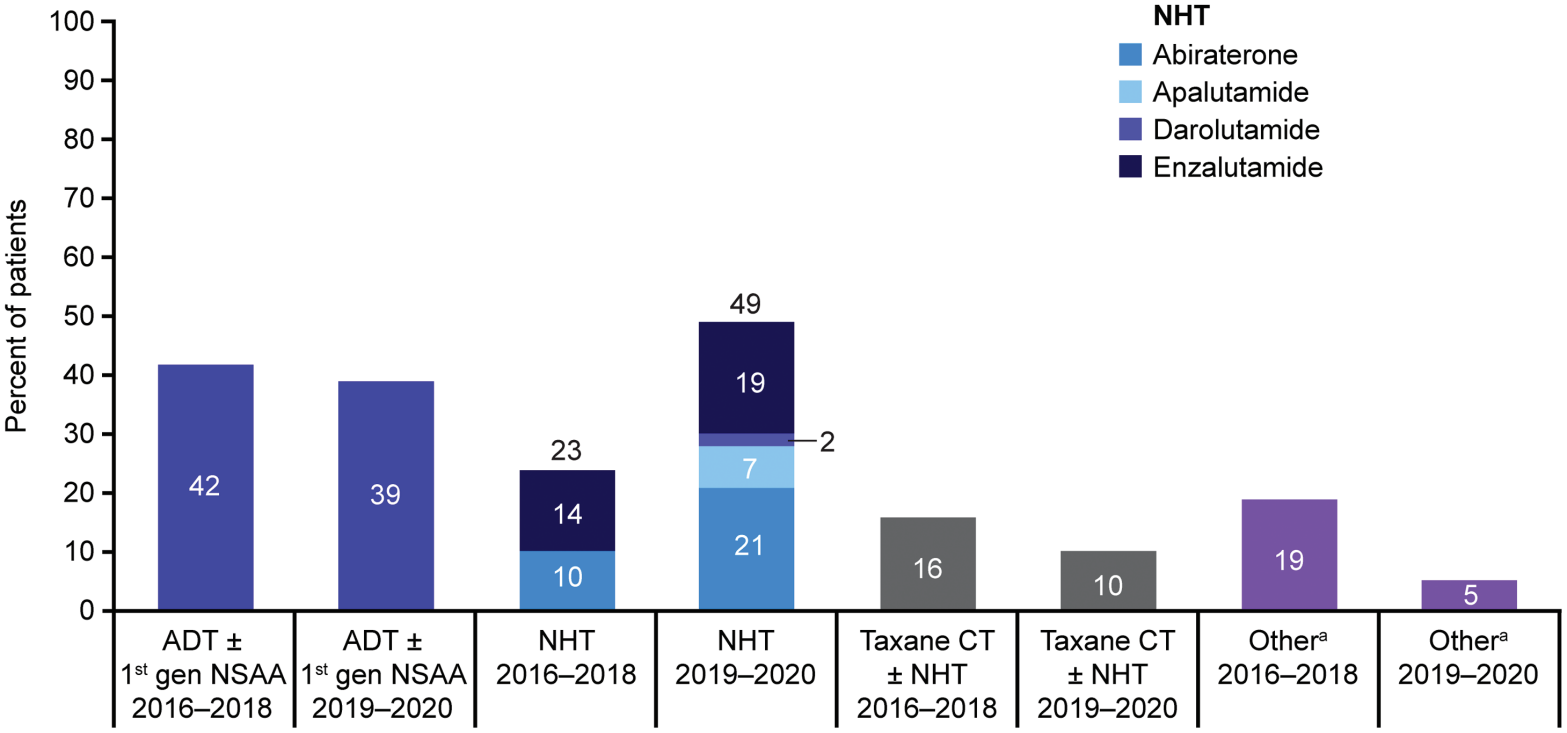
mCSPC treatment trends in the US by year of treatment initiation. mCSPC treatment trends in the US by year of treatment initiation (2016-2018 *n* = 73; 2019-2020 *n* = 168). Percentages may add up to >100% as patients could have had multiple treatment lines initiated within the given time period. No patients in this analysis received darolutamide in combination with docetaxel. ^a^Other treatment intensification included: sipuleucel-T-containing regimen, radium-223-containing regimen, sipuleucel-T- and radium-223-containing regimen, abiraterone + enzalutamide, goserelin + cisplatin + carboplatin, leuprorelin + bicalutamide + docetaxel + carboplatin + prednisone, leuprorelin + mitoxantrone + methylprednisolone, leuprorelin + pembrolizumab, leuprorelin + pembrolizumab + strontium 89 + docetaxel + mitoxantrone. Abbreviations: ADT, androgen deprivation therapy; CT, chemotherapy; gen, generation; mCSPC, metastatic castration-sensitive prostate cancer; NHT, novel hormonal therapy; NSAA, nonsteroidal antiandrogen; US, United States.

In the US, the use of treatment intensification with enzalutamide was 14% and with abiraterone was 10% in 2016-2018. In 2019-2020 the use of treatment intensification with abiraterone was 21%, 19% with enzalutamide, 7% with apalutamide, and 2% with darolutamide. Treatment intensification with taxane chemotherapy was 16% in 2016-2018 and 10% in 2019-2020. Treatment intensification with other agents was 19% in 2016-2018 and 5% in 2019-2020 (Fig. 2). Similar patterns to this were seen for White/Caucasian and African American patients, but greater use of taxane chemotherapy was observed in 2019-2020 than in 2016-2018 for patients of other ethnicities (Supplementary Table S3). When looking at treatment trends by insurance status, more NHT use and less taxane chemotherapy use was observed in 2019-2020 than in 2016-2018 for patients with Medicare and commercial insurance (Supplementary Table S4).

Median (range) time from mCSPC diagnosis to treatment intensification remained at 0 (0-30 and 0-28, respectively) months for 2016-2018 and 2019-2020. Median (range) time from first-line mCSPC treatment initiation to treatment intensification remained at 0 (0-19 and 0-9, respectively) months for 2016-2018 and 2019-2020. Mean (SD) time to treatment intensification following mCSPC diagnosis in 2016-2018 was 3.2 (7.5) months, and in 2019-2020 was 2.5 (6.4) months. Mean (SD) time to treatment intensification following first-line mCSPC treatment initiation in 2016-2018 was 0.4 (2.9) months, and in 2019-2020 was 0.3 (1.5) months (Table 3).

### Treatment Trends Across 5 European Countries and the US by Physician Specialty and Disease Characteristics

In Europe, treatment intensification with a NHT, taxane chemotherapy, or both was 46% for urologist-treated patients and 45% for oncologist-treated patients. In the US, treatment intensification with a NHT was 68% for oncologist-treated patients and 35% for urologist-treated patients (Supplementary Table S5). Disease characteristics across treatment trends are available in Supplementary Table S6.

## Discussion

In concordance with other real-world studies,^32-43^ this study found that most patients with mCSPC do not receive treatment intensification, despite clinical trial evidence^3-18^ and guideline support^2,19-22^ for these therapies. However, our study showed an overall trend toward increasing treatment intensification and decreasing time to treatment intensification across 5 European countries and the US between 2016-2018 and 2019-2020. Of note, to our knowledge, this is one of the first studies to analyze treatment intensification trends in Europe. This is also one of the first studies to investigate treatment intensification trends in both Europe and the US as a single study.

Across 5 European countries, a greater percentage of patients with mCSPC receiving NHT and taxane chemotherapy was seen in 2019-2020 than in 2016-2018. This is reflective of the EMA approval dates of abiraterone (2017),^23^ docetaxel (2019),^24^ and apalutamide (2019).^25^ Enzalutamide was later approved in 2021 for use in patients with mCSPC.^26^

In the US, we observed more NHT use, but less use of taxane chemotherapy and other agents, in 2019-2020 than in 2016-2018. These results are not unexpected given the FDA approval dates of abiraterone (2018),^27^ apalutamide (2019)^28^, and enzalutamide (2019) for mCSPC.^29^ Greater use of abiraterone during 2019-2020 than in 2016-2018 may also be driven by the approval of generic abiraterone in the US during the later time period.^58^ Other US and Canadian claims-based studies also show similar trends for NHT^32,34,36,38-41^ despite covering time periods prior to at least some NHT approval dates. Use of treatment intensification regardless of approval is a trend also seen in our study in both Europe and the US for enzalutamide and docetaxel, respectively.

The period from which our data are drawn overlapped with the COVID-19 pandemic, which likely had an impact on treatment trends. For context, a global survey of 129 healthcare professionals across 17 different countries (including the UK, Spain, and the US) found that 60% of institutions cancelled or postponed systemic anti-cancer therapy for oncology patients due to reasons related to the pandemic (including risk-benefit balance and insufficient staff, capacity, or resources).^59^ All institutions in this study implemented some changes in the delivery of treatment, including 45% of institutions that reported switching patients to oral therapies. In the US, we observed less taxane chemotherapy use and more NHT use in 2019-2020 than in 2016-2018, although in Europe, we observed more taxane chemotherapy and NHT use in 2019-2020 than in 2016-2018. One might speculate that, without the pandemic’s influence, more patients would have received taxane chemotherapy relative to oral treatments such as NHT.

There is some real-world evidence on racial disparities in metastatic prostate cancer in the US that suggests treatment underutilization is of greater concern for Black than White patients.^41,60,61^ In contrast, our study of mCSPC patients in the US found that treatment trends appeared largely similar across ethnicities; more treatment intensification with NHT was observed for all ethnicity groups in the later time period than in the earlier time period. Additionally, although use of ADT ± first-generation NSAA (ie, no treatment intensification), was observed as lower in 2019-2020 than in 2016-2018 for the overall population as well as in African American and other ethnicity patient groups, greater use of this treatment regimen for White/Caucasian patients was observed in 2019-2020 than in 2016-2018. In a US, multi-­institutional, retrospective analysis of 107 Black patients with mCSPC (of whom 87% were diagnosed with stage 4 disease in or after 2015), 27% received ADT ± first-­generation NSAA and 73% received treatment intensification inclusive of 20% ADT + chemotherapy, 45% ADT + NHT, and 8% ADT + chemotherapy + NHT.^62^ Our analysis of US African American patients found similar treatment trends. However, our numbers of patients by US ethnicity were small, so the value of any observations of treatment patterns by ethnicity is limited. Future studies with larger sample sizes across ethnicities should further explore differences in treatment trends.

Our study also investigated how US insurance status affects real-world treatment trends. We found that, while less use of ADT ± first-generation NSAA (ie, no treatment intensification) was observed overall and in patients with Medicare in 2019-2020 than in 2016-2018, greater use of this regimen was observed in 2019-2020 than in 2016-2018 for patients with commercial insurance. The numbers of patients with Medicaid and other/no health insurance were too small to assess.

When looking at the entire study period, treatment intensification rates in Europe appeared similar across physician specialties. However, we observed more treatment intensification overall in oncologist-treated patients than in urologist-treated patients in the US, with no urologists in this study prescribing taxane chemotherapy for their patients with mCSPC.

It is important to recognize that physicians are not the only decision-makers responsible for treatment utilization. Patient preferences based on aspects of their quality-of-life, treatment accessibility, and medical expenses impact the treatments they receive.^48,63^ In a US survey of 100 patients with metastatic prostate cancer, 54% agreed with the statement “Avoiding financial trouble due to treatment of my prostate cancer is very important to me.”^48^ Twenty-one percent of respondents did not agree with the statement “I rely on my doctor to tell me how to treat my prostate cancer.”^48^ A US claims database analysis of commercially insured patients with mCRPC who received treatment intensification found that patients with a low household income were, paradoxically, more likely to receive a more expensive treatment (ie, a NHT).^63^ The authors concluded that qualification for patient assistance programs and thus being shielded from higher out-of-pocket costs may have been a factor in this.

### Limitations

Physician selection in the DSP is a potential bias as it is influenced by willingness to take part and may not be representative of the overall population of physicians treating prostate cancer. Selection also excluded US physicians working at sites such as the Veterans Health Administration, and therefore these patients were not included in this study. Another limitation results from the fact that patients with more frequent visits are more likely to be included in the sample than patients with less frequent visits to their physician. These patients may therefore not fully represent the overall population of patients with mCSPC. However, the systematic approach to recruitment intends to reduce selection bias.

Although the overall sample size for this study was large, sample sizes per country were small; future studies with larger sample sizes per country should investigate this topic further. Additionally, reimbursement for different medications varies widely by country and will therefore influence the treatment trends of each country studied. Reimbursement in European countries may vary by regions within countries; however, this level of analysis was not possible.

Sample size also meant that we were unable to analyze more granular, annual treatment trends; thus, we chose the 2016-2018 and 2019-2020 time periods presented in this study. To be selected for this study, patients with mCSPC during 2016-2018 had to continue medical visits with their physician until 2020, which may have led to differences in baseline characteristics between the 2 time periods. Additionally, sample size is not the same for all patient baseline demographics and disease characteristics. This is because the DSP allowed physicians to state “don’t know” in response to some questions so as not to limit collection.

Our study used descriptive analyses only and we were therefore unable to make conclusions based on comparisons between any patient groups or between the 2 study periods.

## Conclusions

This study found that most patients with mCSPC do not receive treatment intensification despite the availability of these therapies. However, we observed greater use of treatment intensification with both NHT and taxane chemotherapy in 2019-2020 than in 2016-2018 across 5 European countries. In the US, more treatment intensification with NHT was observed for all ethnicity groups, as well as those with Medicare and commercial insurance status, in 2019-2020 than in 2016-2018.

As the number of patients with mCSPC who receive treatment intensification increases and time to treatment intensification decreases, more patients who progress to mCRPC will have been exposed to intensified treatments. As therapies for patients with mCSPC and mCRPC overlap, patients who progress to mCRPC may need new treatment options. This suggests an unmet need will emerge for new therapies in mCRPC and highlights the need for further studies to understand optimal treatment sequencing in mCSPC and mCRPC.

## Supplementary Material

oyad045_suppl_Supplementary_Material

## Data Availability

All data (methodology, materials, data, and data analysis) that support the findings of this survey are the intellectual property of Adelphi Real World. All requests for access should be addressed directly to Amanda Ribbands at amanda.ribbands@adelphigroup.com.

## References

[1] Harada K , Shiota M, Minato A, et al. Treatment strategies for metastatic castration-sensitive prostate cancer: from “All-Comers” to “Personalized” approach. Onco Targets Ther. 2021;14:2967-2974. 10.2147/OTT.S306345. 33981146 PMC8107048

[2] National Comprehensive Cancer Network. Referenced with permission from the NCCN Clinical Practice Guidelines in Oncology (NCCN Guidelines®) for Prostate Cancer V.1.2023. *© National Comprehensive Cancer Network, Inc. 2022. All rights reserved. Available at NCCN.org. Accessed November 14, 2022* [To view the most recent and complete version of the guideline, go online to NCCN.org. NCCN makes no warranties of any kind whatsoever regarding their content, use or application and disclaims any responsibility for their application or use in any way].

[3] Sweeney CJ , Chen YH, Carducci M, et al. Chemohormonal therapy in metastatic hormone-sensitive prostate cancer. N Engl J Med. 2015;373(8):737-746. 10.1056/NEJMoa1503747. 26244877 PMC4562797

[4] Kyriakopoulos CE , Chen YH, Carducci MA, et al. Chemohormonal therapy in metastatic hormone-sensitive prostate cancer: long-term survival analysis of the randomized phase III E3805 CHAARTED trial. J Clin Oncol. 2018;36(11):1080-1087. 10.1200/JCO.2017.75.3657. 29384722 PMC5891129

[5] James ND , Sydes MR, Clarke NW, et al; STAMPEDE investigators. Addition of docetaxel, zoledronic acid, or both to first-line long-term hormone therapy in prostate cancer (STAMPEDE): survival results from an adaptive, multiarm, multistage, platform randomised controlled trial. Lancet. 2016;387(10024):1163-1177. 10.1016/S0140-6736(15)01037-5. 26719232 PMC4800035

[6] Clarke NW , Ali A, Ingleby FC, et al. Addition of docetaxel to hormonal therapy in low- and high-burden metastatic hormone sensitive prostate cancer: long-term survival results from the STAMPEDE trial. Ann Oncol. 2019;30(12):1992-2003. 10.1093/annonc/mdz396. 31560068 PMC6938598

[7] Fizazi K , Tran N, Fein L, et al; LATITUDE Investigators. Abiraterone plus prednisone in metastatic, castration-sensitive prostate cancer. N Engl J Med. 2017;377(4):352-360. 10.1056/NEJMoa1704174. 28578607

[8] Fizazi K , Tran N, Fein L, et al. Abiraterone acetate plus prednisone in patients with newly diagnosed high-risk metastatic castration-sensitive prostate cancer (LATITUDE): final overall survival analysis of a randomised, double-blind, phase 3 trial. Lancet Oncol. 2019;20(5):686-700. 10.1016/S1470-2045(19)30082-8. 30987939

[9] Chi KN , Protheroe A, Rodríguez-Antolín A, et al. Patient-reported outcomes following abiraterone acetate plus prednisone added to androgen deprivation therapy in patients with newly diagnosed metastatic castration-naive prostate cancer (LATITUDE): an international, randomised phase 3 trial. Lancet Oncol. 2018;19(2):194-206. 10.1016/S1470-2045(17)30911-7. 29326030

[10] James ND , de Bono JS, Spears MR, et al; STAMPEDE Investigators. Abiraterone for prostate cancer not previously treated with hormone therapy. N Engl J Med. 2017;377(4):338-351. 10.1056/NEJMoa1702900. 28578639 PMC5533216

[11] James N , Rush H, Clarke N, et al. Abiraterone acetate plus prednisolone for hormone-naïve prostate cancer (PCa): long-term results from metastatic (M1) patients in the STAMPEDE randomised trial (NCT00268476). Ann Oncol. 2020;31(S4):S509. 10.1016/j.annonc.2020.08.871.

[12] Chi KN , Agarwal N, Bjartell A, et al; TITAN Investigators. Apalutamide for metastatic, castration-sensitive prostate cancer. N Engl J Med. 2019;381(1):13-24. 10.1056/NEJMoa1903307. 31150574

[13] Chi KN , Chowdhury S, Bjartell A, et al. Apalutamide in patients with metastatic castration-sensitive prostate cancer: final survival analysis of the randomized, double-blind, phase III TITAN study. J Clin Oncol. 2021;39(20):2294-2303. 10.1200/JCO.20.03488. 33914595

[14] Agarwal N , McQuarrie K, Bjartell A, et al; TITAN investigators. Health-related quality of life after apalutamide treatment in patients with metastatic castration-sensitive prostate cancer (TITAN): a randomised, placebo-controlled, phase 3 study. Lancet Oncol. 2019;20(11):1518-1530. 10.1016/S1470-2045(19)30620-5. 31578173

[15] Davis ID , Martin AJ, Stockler MR, et al; ENZAMET Trial Investigators and the Australian and New Zealand Urogenital and Prostate Cancer Trials Group. Enzalutamide with standard first-line therapy in metastatic prostate cancer. N Engl J Med. 2019;381(2):121-131. 10.1056/NEJMoa1903835. 31157964

[16] Armstrong AJ , Azad AA, Iguchi T, et al. Improved survival with enzalutamide in patients with metastatic hormone-sensitive prostate cancer. J Clin Oncol. 2022;40(15):1616-1622. 10.1200/JCO.22.00193. 35420921 PMC9113211

[17] Armstrong AJ , Szmulewitz RZ, Petrylak DP, et al. ARCHES: a randomized, phase III study of androgen deprivation therapy with enzalutamide or placebo in men with metastatic hormone-sensitive prostate cancer. J Clin Oncol. 2019;37(32):2974-2986. 10.1200/JCO.19.00799. 31329516 PMC6839905

[18] Stenzl A , Szmulewitz RZ, Petrylak D, et al. The impact of enzalutamide on quality of life in men with metastatic hormone-­sensitive prostate cancer based on prior therapy, risk, and symptom ­subgroups. Prostate. 2022; 10.1002/pros.24396: 10.1002/pros.24396. Online ahead of print. 35675470

[19] Lowrance WT , Breau RH, Chou R, et al. Advanced prostate cancer: AUA/ASTRO/SUO guideline part I. J Urol. 2021;205(1):14-21. 10.1097/JU.0000000000001375. 32960679

[20] Virgo KS , Rumble RB, de Wit R, et al. Initial management of noncastrate advanced, recurrent, or metastatic prostate cancer: ASCO guideline update. J Clin Oncol. 2021;10(11):1274-1305. https://doi.org/10.1200/JCO.20.03256. 10.1200/JCO.20.03256 33497248

[21] Parker C , Castro E, Fizazi K, et al; ESMO Guidelines Committee. Electronic address: clinicalguidelines@esmo.org. Prostate cancer: ESMO clinical practice guidelines for diagnosis, treatment and follow-up. Ann Oncol. 2020;31(9):1119-1134. 10.1016/j.annonc.2020.06.011. 32593798

[22] Mottet N , Cornford P, van den Bergh RCN, et al. *EAU-EANM-ESTRO-ESUR-SIOG guidelines on prostate cancer*. Accessed August 18, 2022, https://d56bochluxqnz.cloudfront.net/documents/full-guideline/EAU-EANM-ESTRO-ESUR-ISUP_SIOG-Guidelines-on-Prostate-Cancer-2022_2022-04-25-063938_yfos.pdf.

[23] ESMO Oncology News. *EMA recommends extension of indications for abiraterone acetate*. Accessed April 5, 2022, https://www.esmo.org/oncology-news/archive/ema-recommends-extension-of-indications-for-abiraterone-acetate.

[24] ESMO Oncology News. *EMA recommends extension of indications for taxotere and docetaxel zentiva*. Accessed April 5, 2022, https://www.esmo.org/oncology-news/EMA-Recommends-Extension-of-Indications-for-Taxotere-and-Docetaxel-Zentiva.

[25] ESMO Oncology News. *EMA recommends extension of therapeutic indications for apalutamide*. Accessed April 5, 2022, https://www.esmo.org/oncology-news/ema-recommends-extension-of-therapeutic-indications-for-apalutamide.

[26] ESMO Oncology News. *EMA recommends extension of indications for enzalutamide*. Accessed April 5, 2022, https://www.esmo.org/oncology-news/ema-recommends-extension-of-indications-for-enzalutamide.

[27] U.S. Food & Drug Administration. *FDA approves abiraterone acetate in combination with prednisone for high-risk metastatic castration-sensitive prostate cancer*. Accessed April 5, 2022, https://www.fda.gov/drugs/resources-information-approved-drugs/fda-approves-abiraterone-acetate-combination-prednisone-high-risk-metastatic-castration-sensitive.

[28] U.S. Food & Drug Administration. *FDA approves apalutamide for metastatic castration-sensitive prostate cancer*. Accessed April 5, 2022, https://www.fda.gov/drugs/resources-information-approved-drugs/fda-approves-apalutamide-metastatic-castration-sensitive-prostate-cancer.

[29] U.S. Food & Drug Administration. *FDA approves enzalutamide for metastatic castration-sensitive prostate cancer*. Accessed April 5, 2022, https://www.fda.gov/drugs/resources-information-approved-drugs/fda-approves-enzalutamide-metastatic-castration-sensitive-prostate-cancer.

[30] U.S. Food & Drug Administration. *Taxotere Prescribing Information*. Accessed April 5, 2022, https://www.accessdata.fda.gov/drugsatfda_docs/label/2010/020449s059lbl.pdf.

[31] Saiyed MM , Ong PS, Chew L. Off-label drug use in oncology: a systematic review of literature. J Clin Pharm Ther. 2017;42(3):251-258. 10.1111/jcpt.12507. 28164359

[32] Freedland SJ , Sandin R, Sah J, et al. Treatment patterns and survival in metastatic castration-sensitive prostate cancer in the US Veterans health administration. Cancer Med. 2021;10(23):8570-8580. 10.1002/cam4.4372. 34725947 PMC8633245

[33] Ryan CJ , Ke X, Lafeuille MH, et al. Management of patients with metastatic castration-sensitive prostate cancer in the real-world setting in the United States. J Urol. 2021;206(6):1420-1429. 10.1097/JU.0000000000002121. 34293915 PMC8584208

[34] Swami U , Sinnott JA, Haaland B, et al. Treatment pattern and outcomes with systemic therapy in men with metastatic prostate cancer in the real-world patients in the United States. Cancers (Basel). 2021;13(19):4951. 34638435 10.3390/cancers13194951 PMC8508241

[35] Wallis CJ , Malone S, Cagiannos I, et al. Real-world use of ­androgen-deprivation therapy: intensification among older Canadian men with de novo metastatic prostate cancer. JNCI Cancer Spectr. 2021;5(6):pkab082. 34926988 10.1093/jncics/pkab082 PMC8678925

[36] Swami U , Hong A, El-Chaar NN, et al. Real-world first-line (1L) treatment patterns in patients (pts) with metastatic castration-­sensitive prostate cancer (mCSPC) in a U.S. health insurance database. J Clin Oncol. 2021;39(15_suppl):5072-5072. 10.1200/jco.2021.39.15_suppl.5072.

[37] Tagawa ST , Sandin R, Sah J, Mu Q, Freedland SJ. 679P Treatment patterns of metastatic castration-sensitive prostate cancer (mCSPC): a real-world evidence study. Ann Oncol. 2020;31:S541-S542. 10.1016/j.annonc.2020.08.938.

[38] Freedland SJ , Sandin R, Tagawa ST, et al. 609P Treatment patterns and overall survival (OS) in metastatic castration-sensitive prostate cancer (mCSPC) from 2006 to 2019. Ann Oncol. 2021;32:S650-S651. 10.1016/j.annonc.2021.08.1122.

[39] George DJ , Agarwal N, Ramaswamy K, et al. 616P Real-world utilization of advanced therapies by metastatic site and age among patients with metastatic castration-­sensitive prostate cancer (mCSPC): a Medicare database analysis. Ann Oncol. 2021;32:S655-S656. 10.1016/j.annonc.2021.08.1129.

[40] George DJ , Agarwal N, Rider JR, et al. Real-world treatment patterns among patients diagnosed with metastatic castration-sensitive prostate cancer (mCSPC) in community oncology settings. J Clin Oncol. 2021;39(15_suppl):5074-5074. 10.1200/jco.2021.39.15_suppl.5074.

[41] Freedland SJ , Agarwal N, Ramaswamy K, et al. Real-world utilization of advanced therapies and racial disparity among patients with metastatic castration-sensitive prostate cancer (mCSPC): a Medicare database analysis. J Clin Oncol. 2021;39(15_suppl):5073-5073. 10.1200/jco.2021.39.15_suppl.5073.

[42] Swami U , Hong A, El-Chaar NN, et al. Underutilization of standard of care (SOC) treatment intensification in patients (pts) with metastatic castration-sensitive prostate cancer (mCSPC) by specialty. J Clin Oncol. 2022;40(6_suppl):183-183. 10.1200/jco.2022.40.6_suppl.183.

[43] Yip S , Niazi T, Hotte SJ, et al. Evolving real-world patterns of practice in metastatic castration-sensitive prostate cancer (mCSPC): the genitourinary research consortium (GURC) national multicenter cohort study. J Clin Oncol. 2022;40(6_suppl):86-86. 10.1200/jco.2022.40.6_suppl.086.

[44] Fizazi K , Carles Galceran J, Foulon S, et al. LBA5 A phase III trial with a 2x2 factorial design in men with de novo metastatic ­castration-sensitive prostate cancer: overall survival with abiraterone acetate plus prednisone in PEACE-1. Ann Oncol. 2021;32:S1299. 10.1016/j.annonc.2021.08.2099.

[45] Smith MR , Hussain M, Saad F, et al; ARASENS Trial Investigators. Darolutamide and survival in metastatic, hormone-sensitive prostate cancer. N Engl J Med. 2022;386(12):1132-1142. 10.1056/NEJMoa2119115. 35179323 PMC9844551

[46] Cheema PK , Gavura S, Migus M, et al. International variability in the reimbursement of cancer drugs by publically funded drug programs. Curr Oncol. 2012;19(3):165-176. 10.3747/co.19.946 PMC3364777 22670106

[47] Nolte E , Corbett J. International variation in drug usage: an exploratory analysis of the “Causes” of variation. Rand Health Q. 2015;4(4):1. 28083348 10.7249/rhq-04-04-01 PMC5158257

[48] Oswald LB , Schumacher FA, Gonzalez BD, et al. What do men with metastatic prostate cancer consider when making treatment decisions? A mixed-methods study. Patient Prefer Adherence. 2020;14:1949-1959. 10.2147/PPA.S271620. 33116438 PMC7569052

[49] Schumacher FA , Helenowski IB, Oswald LB, et al. Treatment ­decision-making in metastatic prostate cancer: perceptions of locus of control among patient, caregiver, and physician triads. Patient Prefer Adherence. 2022;16:235-244. 10.2147/PPA.S334827. 35125865 PMC8811793

[50] Freedland SJ , Klaassen ZWA, Agarwal N, et al. Reasons for ­oncologist and urologist treatment choice in metastatic ­castration-sensitive prostate cancer (mCSPC): a physician survey linked to patient chart reviews in the United States. J Clin Oncol. 2022;40(16_suppl):5065-5065. 10.1200/jco.2022.40.16_suppl.5065.

[51] Anderson P , Benford M, Harris N, Karavali M, Piercy J. Real-world physician and patient behaviour across countries: disease-specific programmes—a means to understand. Curr Med Res Opin. 2008;24(11):3063-3072. 10.1185/03007990802457040. 18826746

[52] Babineaux SM , Curtis B, Holbrook T, Milligan G, Piercy J. Evidence for validity of a national physician and patient-reported, cross-sectional survey in China and UK: the disease specific programme. BMJ Open. 2016;6(8):e010352. 10.1136/bmjopen-2015-010352. PMC5013497 27531722

[53] Higgins V , Piercy J, Roughley A, et al. Trends in medication use in patients with type 2 diabetes mellitus: a long-term view of real-world treatment between 2000 and 2015. Diabetes Metab Syndr Obes. 2016;9:371-380. 10.2147/DMSO.S120101. 27843332 PMC5098530

[54] ECOG-ACRIN Cancer Research Group. *ECOG Performance Status Scale*. Accessed May 19, 2022, https://ecog-acrin.org/resources/ecog-performance-status/.

[55] European Pharmaceutical Market Research Association (EphMRA). *Code of Conduct*. Accessed August 18, 2022, https://www.ephmra.org/code-conduct-aer.

[56] US Department of Health and Human Services. *Summary of the HIPAA Privacy Rule*. Accessed August 18, 2022, http://www.hhs.gov/sites/default/files/privacysummary.pdf.

[57] Health Information Technology (HITECH). *Health Information Technology Act*. Accessed August 18, 2022, https://www.hhs.gov/hipaa/for-professionals/special-topics/hitech-act-enforcement-interim-final-rule/index.html.

[58] Novadoz. *MSN Labs/Novadoz Pharmaceuticals Early Success Continues With FDA Approval of Generic Abiraterone*. Accessed April 5, 2022, http://novadozpharma.com/news/.

[59] Chow MC , Chambers P, Singleton G, et al. Global changes to the chemotherapy service during the covid-19 pandemic. J Oncol Pharm Pract. 2021;27(5):1073-1079. 10.1177/10781552211015767. 33983080 PMC8367193

[60] Smith MR , Saad F, Chowdhury S, et al. Apalutamide and overall survival in prostate cancer. Eur Urol. 2021;79(1):150-158. 10.1016/j.eururo.2020.08.011. 32907777

[61] George DJ , Ramaswamy K, Huang A, et al. Survival by race in men with chemotherapy-naive enzalutamide- or abiraterone-treated metastatic castration-resistant prostate cancer. Prostate Cancer Prostatic Dis. 2022;25(3):524-530. 10.1038/s41391-021-00463-9. 34732856 PMC9385484

[62] Freeman MN , Jang A, Zhu J, et al. Multi-institutional analysis of the clinical and genomic characteristics of black patients with metastatic hormone-sensitive prostate cancer. Oncologist. 2022;27(3):220-227. 10.1093/oncolo/oyab057. 35274720 PMC8914485

[63] Caram MEV , Wang S, Tsao P, et al. Patient and provider variables associated with variation in the systemic treatment of advanced prostate cancer. Urol Pract. 2019;6(4):234-242. 10.1097/UPJ.0000000000000020. 31276025 PMC6605774

