# Supplementary material for: Real-World Treatment Trends Among Patients with Metastatic Castration-Sensitive Prostate Cancer: Results from an International Study
Source: Oncologist. 2023 Apr 4;28(9):780–9. doi: 10.1093/oncolo/oyad045 (PMC10485292; doi:10.1093/oncolo/oyad045)
Supplement: oyad045_suppl_Supplementary_Material [file oyad045_suppl_supplementary_material.docx]

Supplementary Table S1A. Patient demographics by country (Europe).

|  | **Country** | | | | |
| --- | --- | --- | --- | --- | --- |
|  | **UK**  **(*n* = 200)** | **France**  **(*n* = 378)** | **Germany (*n* = 266)** | **Spain**  **(*n* = 222)** | **Italy**  **(*n* = 255)** |
| **Physician specialty, *n* (%)** | | | | | |
| Oncologist | 187 (94) | 306 (81) | 175 (66) | 188 (85) | 255 (100) |
| *Medical oncologist* | 115 (58) | 306 (81) | 168 (63) | 182 (82) | 246 (96) |
| *Radiation oncologist* | 4 (2) | 0 (0) | 7 (3) | 6 (3) | 9 (4) |
| *Clinical oncologist^a^* | 68 (34) | 0 (0) | 0 (0) | 0 (0) | 0 (0) |
| Urologist^b^ | 13 (7) | 72 (19) | 91 (34) | 34 (15) | 0 (0) |
| **Hospital type, *n* (%)** |  |  |  |  |  |
| Academic/cancer center | 155 (78) | 214 (57) | 51 (19) | 192 (86) | 93 (36) |
| Community | 45 (23) | 164 (43) | 215 (81) | 30 (14) | 162 (64) |
| **Patient age at time of data collection, *n* (%)** | | | | | |
| Median (range) | 72.0  (45–90) | 74.0  (50–90) | 70.0  (54–88) | 72.0  (48–90) | 74.0  (49–90) |
| **Patient age at time of mCSPC diagnosis, *n* (%)** | | | | | |
| Total | 91 | 163 | 127 | 86 | 119 |
| Median (range) | 71.0  (44–83) | 70.0  (51–86) | 69.0  (53–85) | 70.0  (53–87) | 73.0  (47–88) |
| **Family history of prostate cancer, *n* (%)** | | | | | |
| Yes | 15 (8) | 35 (9) | 42 (16) | 25 (11) | 13 (5) |
| No | 174 (87) | 331 (88) | 211 (79) | 186 (84) | 220 (86) |
| Don't know | 11 (6) | 12 (3) | 13 (5) | 11 (5) | 22 (9) |

^a^Clinical oncologist is a UK-specific specialty covering both medical and radiation specialties.

^b^Urologist includes seven physicians in Spain who selected “Prostate/specialist cancer surgeon.”

Abbreviations: mCSPC, metastatic castration-sensitive prostate cancer; UK, United Kingdom.

Supplementary Table S1B. Patient clinical characteristics by country (Europe).

|  | **Country** | | | | |
| --- | --- | --- | --- | --- | --- |
|  | **UK**  **(*n* = 200)** | **France**  **(*n* = 378)** | **Germany (*n* = 266)** | **Spain**  **(*n* = 222)** | **Italy**  **(*n* = 255)** |
| **Disease state at time of data collection, *n* (%)** | | | | | |
| mCSPC | 116 (58) | 238 (63) | 178 (67) | 157 (71) | 160 (63) |
| mCRPC with mCSPC treatment history | 84 (42) | 140 (37) | 88 (33) | 65 (29) | 95 (37) |
| **Patients with metastases at time of data collection, *n* (%)** | | | | | |
| Bone | 183 (92) | 358 (95) | 195 (73) | 206 (93) | 221 (87) |
| Non-regional / distant lymph nodes | 59 (30) | 129 (34) | 96 (36) | 94 (42) | 103 (40) |
| Visceral | 32 (16) | 75 (20) | 53 (20) | 46 (21) | 50 (20) |
| *Liver* | 17 (9) | 35 (9) | 15 (6) | 19 (9) | 10 (4) |
| Other | 2 (1) | 5 (1) | 1 (0) | 2 (1) | 0 (0) |
| **ECOG score at mCSPC diagnosis, *n* (%)** | | | | | |
| 0 | 47 (24) | 86 (23) | 74 (28) | 55 (25) | 91 (36) |
| 1 | 129 (65) | 213 (56) | 147 (55) | 136 (61) | 116 (45) |
| 2 | 19 (10) | 73 (19) | 43 (16) | 24 (11) | 32 (13) |
| 3 | 5 (3) | 6 (2) | 2 (1) | 7 (3) | 13 (5) |
| 4 | 0 (0) | 0 (0) | 0 (0) | 0 (0) | 2 (1) |
| Unknown / not assessed | 0 (0) | 0 (0) | 0 (0) | 0 (0) | 1 (0) |

Abbreviations: ECOG, Eastern Cooperative Oncology Group; mCSPC, metastatic castration-sensitive prostate cancer; mCRPC, metastatic castration-resistant prostate cancer; UK, United Kingdom.

Supplementary Table S2. US insurance status by ethnicity.

|  | **Ethnicity** | | | |
| --- | --- | --- | --- | --- |
|  | **White/ Caucasian**  **(*n* = 159)** | **African American**  **(*n* = 49)** | **Other**^a^  **(*n* = 31)** | **Total (All Ethnicities)**  **(*n* = 239)** |
| **Insurance status, *n* (%)** | |  |  |  |
| Medicaid | 7 (4) | 4 (8) | 4 (13) | 15 (6) |
| Medicare | 88 (55) | 34 (69) | 17 (55) | 139 (58) |
| Commercial | 62 (39) | 7 (14) | 9 (29) | 78 (33) |
| Other/no health insurance | 2 (1) | 4 (8) | 1 (3) | 7 (3) |

^a^Other ethnicities include Hispanic/Latino, Asian, Middle Eastern, and mixed race.

Abbreviation: US, United States.

Supplementary Table S3. First-line mCSPC treatment trends in the US by ethnicity.

| **First-line mCSPC treatment, *n* (%)** | **2016–2018** | | | **2019–2020** | | | **Total (2016–2020)** | | |
| --- | --- | --- | --- | --- | --- | --- | --- | --- | --- |
|  | **White/ Caucasian**  **(n = 46)** | **African American**  **(n = 15)** | **Other**^a^  **(n = 12)** | **White/ Caucasian**  **(n = 115)** | **African American**  **(n = 34)** | **Other**^a^  **(n = 19)** | **White/ Caucasian**  **(n = 159)** | **African American**  **(n = 49)** | **Other**^a^  **(n = 31)** |
| ADT ± first-generation NSAA | 17 (37) | 6 (40) | 8 (67) | 48 (42) | 10 (29) | 8 (42) | 65 (41) | 16 (33) | 16 (52) |
| ADT + NHT | 10 (22) | 4 (27) | 2 (17) | 51 (44) | 18 (53) | 6 (32) | 61 (38) | 22 (45) | 8 (26) |
| ADT + taxane chemotherapy ± NHT | 8 (17) | 4 (27) | 0 (0) | 10 (9) | 4 (12) | 3 (16) | 17 (11) | 8 (16) | 3 (10) |
| ADT + other^b^ | 11 (24) | 1 (7) | 2 (17) | 6 (5) | 2 (6) | 2 (11) | 16 (10) | 3 (6) | 4 (13) |

^a^Other ethnicities include: Hispanic/Latino, Asian, Middle Eastern, mixed race.

^b^Other treatment intensification includes: sipuleucel-T-containing regimen, radium-223-containing regimen, sipuleucel-T- and radium-223-containing regimen, abiraterone + enzalutamide, goserelin + cisplatin + carboplatin, leuprorelin + bicalutamide + docetaxel + carboplatin + prednisone, leuprorelin + mitoxantrone + methylprednisolone, leuprorelin + pembrolizumab, leuprorelin + pembrolizumab + strontium 89 + docetaxel + mitoxantrone.

Abbreviations: ADT, androgen deprivation therapy; mCSPC, metastatic castration-sensitive prostate cancer; NHT, novel hormonal therapy; NSAA, nonsteroidal antiandrogen; US, United States.

Supplementary Table S4. mCSPC treatment trends in the US by insurance status.

| **First-line mCSPC treatment, *n* (%)** | **2016–2018** | | | | **2019–2020** | | | | **Total (2016–2020)** | | | |
| --- | --- | --- | --- | --- | --- | --- | --- | --- | --- | --- | --- | --- |
|  | **Medicare**  **(*n* = 52)** | **Commercial**  **(*n* = 14)** | **Medicaid**  **(*n* = 5)** | **Other/no health insurance**  **(*n* = 2)** | **Medicare**  **(*n* = 88)** | **Commercial**  **(*n* = 65)** | **Medicaid**  **(*n* = 10)** | **Other/no health insurance**  **(*n* = 5)** | **Medicare**  **(*n* = 139)** | **Commercial**  **(*n* = 78)** | **Medicaid**  **(*n* = 15)** | **Other/no health insurance**  **(*n* = 7)** |
| ADT ± first-generation NSAA | 24 (46) | 3 (21) | 4 | 0 | 29 (33) | 33 (51) | 2 | 2 | 53 (38) | 36 (46) | 6 | 2 |
| ADT + NHT | 11 (21) | 4 (29) | 0 | 1 | 46 (52) | 23 (35) | 4 | 2 | 57 (41) | 27 (35) | 4 | 3 |
| ADT + taxane chemotherapy ± NHT | 10 (19) | 2 (14) | 0 | 0 | 8 (9) | 6 (9) | 2 | 1 | 17 (12) | 8 (10) | 2 | 1 |
| ADT + other^a^ | 7 (13) | 5 (36) | 1 | 1 | 5 (6) | 3 (5) | 2 | 0 | 12 (9) | 7 (9) | 3 | 1 |

^a^Other treatment intensification includes: sipuleucel-T-containing regimen, radium-223-containing regimen, sipuleucel-T- and radium-223-containing regimen, abiraterone + enzalutamide, goserelin + cisplatin + carboplatin, leuprorelin + bicalutamide + docetaxel + carboplatin + prednisone, leuprorelin + mitoxantrone + methylprednisolone, leuprorelin + pembrolizumab, leuprorelin + pembrolizumab + strontium 89 + docetaxel + mitoxantrone.

Abbreviations: ADT, androgen deprivation therapy; mCSPC, metastatic castration-sensitive prostate cancer; NHT, novel hormonal therapy; NSAA, nonsteroidal antiandrogen; US, United States.

**Supplementary Table S5.** First-line mCSPC treatment trends by physician specialty across five European countries and the US (2016–2020).

| **First-line mCSPC treatment, *n* (%)** | **Europe** | | **US** | |
| --- | --- | --- | --- | --- |
|  | **Physician specialty** | | | |
|  | **Oncologist**  **(*n* = 1111)** | **Urologist**  **(*n* = 210)** | **Oncologist**  **(*n* = 179)** | **Urologist**  **(*n* = 60)** |
| ADT ± first-generation NSAA | 610 (55) | 112 (53) | 58 (32) | 39 (65) |
| ADT + NHT | 245 (22) | 59 (28) | 80 (45) | 11 (18) |
| ADT + taxane chemotherapy ± NHT | 254 (23) | 38 (18) | 28 (16) | 0 (0) |
| ADT + other^a^ | 2 (0) | 1 (0) | 13 (7) | 10 (17) |

^a^Other treatment intensification in Europe includes radium-223; other treatment intensification in the US includes sipuleucel-T-containing regimen, radium-223-containing regimen, sipuleucel-T- and radium-223-containing regimen, abiraterone + enzalutamide, goserelin + cisplatin + carboplatin, leuprorelin + bicalutamide + docetaxel + carboplatin + prednisone, leuprorelin + mitoxantrone + methylprednisolone, leuprorelin + pembrolizumab, leuprorelin + pembrolizumab + strontium 89 + docetaxel + mitoxantrone.

Abbreviations: ADT, androgen deprivation therapy; mCSPC, metastatic castration-sensitive prostate cancer; NHT, novel hormonal therapy; NSAA, nonsteroidal antiandrogen; US, United States.

**Supplementary Table S6.** Disease characteristics by first-line mCSPC treatment trends in five European countries and the US (2016–2020).

|  | **First-line mCSPC treatment (Europe and US)** | | | | |
| --- | --- | --- | --- | --- | --- |
|  | **ADT ± first-generation NSAA**  **(*n* = 819)** | **ADT + NHT**  **(*n* = 395)** | **ADT + taxane chemotherapy ± NHT**  **(*n* = 320)** | **ADT + other**^a^  **(*n* = 26)** | **Total**  **(*n* = 1560)** |
| **Disease state at time of data collection, *n* (%)** | | | | | |
| mCSPC | 442 (54) | 310 (78) | 226 (71) | 17 (65) | 995 (64) |
| mCRPC with mCSPC treatment history | 377 (46) | 85 (22) | 94 (29) | 9 (35) | 565 (36) |
| **Patients with metastases at time of data collection, *n* (%)** | | | | | |
| Bone | 693 (85) | 334 (85) | 280 (88) | 20 (77) | 1327 (85) |
| Non-regional / distant lymph nodes | 273 (33) | 148 (37) | 119 (37) | 7 (27) | 547 (35) |
| Visceral | 125 (15) | 98 (25) | 99 (31) | 5 (19) | 327 (21) |
| *Liver* | 28 (3) | 34 (9) | 49 (15) | 1 (4) | 112 (7) |
| Other | 6 (1) | 1 (0) | 7 (2) | 0 (0) | 14 (1) |
| **Disease volume at time of data collection, *n* (%)** | | | | | |
| Low | 425 (52) | 179 (45) | 113 (35) | 9 (35) | 726 (47) |
| High | 261 (32) | 171 (43) | 182 (57) | 12 (46) | 626 (40) |
| Don't Know | 133 (16) | 45 (11) | 25 (8) | 5 (19) | 208 (13) |
| **ECOG score at mCSPC diagnosis, *n* (%)** | | | | | |
| 0 | 235 (29) | 84 (21) | 82 (26) | 12 (46) | 413 (26) |
| 1 | 422 (52) | 258 (65) | 200 (63) | 11 (42) | 891 (57) |
| 2 | 133 (16) | 45 (11) | 34 (11) | 3 (12) | 215 (14) |
| 3 | 26 (3) | 5 (1) | 4 (1) | 0 (0) | 35 (2) |
| 4 | 2 (0) | 2 (1) | 0 (0) | 0 (0) | 4 (0) |
| Unknown / not assessed | 1 (0) | 1 (0) | 0 (0) | 0 (0) | 2 (0) |

^a^Other treatment intensification includes sipuleucel-T-containing regimen, radium-223-containing regimen, sipuleucel-T- and radium-223-containing regimen, abiraterone + enzalutamide, goserelin + cisplatin + carboplatin, leuprorelin + bicalutamide + docetaxel + carboplatin + prednisone, leuprorelin + mitoxantrone + methylprednisolone, leuprorelin + pembrolizumab, leuprorelin + pembrolizumab + strontium 89 + docetaxel + mitoxantrone.

Abbreviations: ADT, androgen deprivation therapy; mCSPC, metastatic castration-sensitive prostate cancer; NHT, novel hormonal therapy; NSAA, nonsteroidal antiandrogen; US, United States.
